# Systematic analysis of RNASET2 gene as a potential prognostic and immunological biomarker in clear cell renal cell carcinoma

**DOI:** 10.1186/s12885-023-11356-6

**Published:** 2023-09-07

**Authors:** Yifu Liu, Zhicheng Zhang, Ping Xi, Ru Chen, Xiaofeng Cheng, Ji Liu, Qiqi Zhu, Yechen Nie, Ting Sun, Binbin Gong, Siyuan Wang

**Affiliations:** 1 Department of Urology, The First Affiliated Hospital of Nanchang University, No.17, Yongwai Center Street, Donghu District, Nanchang, 330006 Jiangxi China; 2 Department of Urology, Sichuan Cancer Hospital School of Medicine, University of Electronic Science and Technology of China, No. 55, Section 4, Renmin South Road, Chengdu, 610041 Sichuan China

**Keywords:** RNASET2, Clear cell renal cell carcinoma, Prognostic, Biomarker

## Abstract

**Background:**

RNASET2 has been identified as an oncogene with anti-angiogenic and immunomodulatory effects in a variety of cancers, but its function in clear cell renal cell carcinoma (ccRCC) is still not well understood.

**Methods:**

The RNASET2 expression matrix was extracted from the The Cancer Genome Atlas (TCGA) and Gene Expression Omnibus (GEO) datasets and analyzed for diagnostic and prognostic value. RNASET2 mRNA expression was detected by quantitative polymerase chain reaction (qPCR) in ccRCC patients and renal cancer cell lines. Wound healing assay, transwell assay, western blotting, and tube formation assays were used to evaluate the function of RNASET2 in renal cancer in vitro. In addition, transcriptome sequencing was performed on knockdown RNASET2 kidney cancer cells to analyze their potential signaling pathways. Moreover, the immune microenvironment and mutational status were evaluated to predict the potential mechanisms of RNASET2 involvement in renal cancer progression. Sensitivity to common chemotherapeutic and targeted agents was assessed according to the Genomics of Drug Sensitivity in Cancer (GDSC) database.

**Results:**

RNASET2 expression was significantly upregulated in ccRCC tissues and renal cancer cell lines, predicting poor prognosis for patients. In vitro experiments showed that silencing RNASET2 inhibited the migration and pro-angiogenic ability of renal cancer cells. Transcriptome sequencing suggested its possible involvement in the remodeling of the immune microenvironment in renal cell carcinoma. Furthermore, bioinformatics analysis and immunohistochemical staining showed that RNASET2 was positively correlated with the infiltration abundance of regulatory T cells. Finally, we mapped the mutational landscape of RNASET2 in ccRCC and found its predictive value for drug sensitivity.

**Conclusions:**

Our results suggest that RNASET2 is a promising biomarker and therapeutic target in ccRCC.

**Supplementary Information:**

The online version contains supplementary material available at 10.1186/s12885-023-11356-6.

## Introduction

Renal cell carcinoma (RCC) is characterized by malignant tumors originating from the epithelial cells of the renal tubules, which are highly heterogeneous [1]. Overall, approximately 400,000 people are newly diagnosed with RCC worldwide each year and more than 150,000 people die from the disease [2]. The disease can be divided into various histological subtypes, with clear cell renal cell carcinoma (ccRCC) being the most prevalent in the population (over 70%). Although early ccRCC patients can achieve satisfactory clinical outcomes with surgical or radiofrequency ablation strategies, the fact remains that the proportion of such patients remains low in the real world, especially in economically disadvantaged areas, due to the lack of significant clinical presentation [3]. Unfortunately, more ccRCC patients will inevitably experience metastasis of the lesion during his lifetime. And, for patients with ccRCC at this stage, the 5-year cumulative survival rate is only 11.7%.

It is well known that conventional chemotherapy and radiotherapy strategies are largely ineffective for patients with advanced ccRCC. Encouragingly, due to the vascular-rich and immune-infiltrating nature of ccRCC, targeted anti-angiogenic therapies, immune checkpoint inhibitors, or combination therapies have undoubtedly become the first-line treatment option, with good results [4]. However, given the heterogeneity of tumors and the resistance of drugs, more potential targets still need to be explored to refine the molecular and immune landscape of ccRCC [5].

Ribonuclease T2 (RNASET2) is the first member of the Rh/T2/S glycoprotein family to be identified in humans and is present in multiple forms in human cell lines [6]. The gene was found to be localized to chromosome 6q27, which is deleted and rearranged in several human cancers, suggesting an association with tumorigenesis [7–10]. For example, in ovarian cancer, gastric adenocarcinoma and melanoma, downregulation of RNASET2 significantly enhanced the malignant phenotype of the tumor and correlated with a worsening of the patient’s clinical outcome [11–13]. Also, evidence suggests that recombinant human RNASET2 glycoprotein possesses anti-tumor and anti-angiogenic properties in both in vivo and in vitro assays [14]. Taken together, this evidence supports the close association of RNASET2 with human cancer from multiple perspectives.

Nevertheless, to our knowledge, the role of RNASET2 in ccRCC remains unrevealed. Therefore, based on bioinformatics and in vitro experiments, we performed a preliminary exploration of the biological function of RNASET2 in ccRCC in the expectation of providing ideas for finding new therapeutic targets.

## Materials and methods

### Public data access

RNA sequencing matrices of ccRCC tissues and normal kidney tissues were downloaded from the The Cancer Genome Atlas (TCGA) database (http://tcga-data.nci.nih.gov/tcga) and Gene Expression Omnibus (GEO) database (https://www.ncbi.nlm.nih.gov/gds/) to analyze the mRNA expression levels of RNASET2, and the Human Protein Atlas (HPA) database (https://www.proteinatlas.org/) was used to compare its protein expression in the tissues [15]. Immunological characterization of RNASET2 was obtained from TIMER2.0 (http://timer.cistrome.org/) and the TISIDB database (http://cis.hku.hk/TISIDB/) [16, 17].The mutation type of RNASET2 in ccRCC was assessed using the Catalogue of Somatic Mutations in Cancer (COSMIC) database(https://cancer.sanger.ac.uk/cosmic) [18, 19].

### Clinical tissue samples and cell lines

Eight pairs of ccRCC tissues and paracancerous tissues were obtained from the patients’ postoperative specimens. Human renal tubular epithelial cells (HK-2) and kidney cell carcinoma cells (A498, 786-O, OSRC-2) were purchased from the Chinese Academy of Sciences and cultured in Dulbecco’s modified Eagle medium-high glucose (DMEM-H, Gibco) medium, modified Eagle medium (MEM, Boster) and RPMI 1640 (Gibco) and with 10% fetal bovine serum (FBS, Vivacell) as well as 1% penicillin-streptomycin, respectively. The human umbilical vein endothelial cells (HUVECs) were purchased from the Shanghai Institute of Cell Biology, Chinese Academy of Sciences, and cultured in Dulbecco’s modified Eagle medium (DMEM, Gibco) with 15% FBS, together with 1% penicillin and streptomycin. All of these cells were cultured in a 37 °C, 5% CO2 thermostat.

### Quantitative real-time PCR

After extraction of total RNA from cell lines and 8 pairs clinical tissue samples, reverse transcription and PCR were performed using cDNA synthesis kits (TransGen, China) and SYBR real-time PCR kits (TransGen, China), respectively, under the manufacturer’s instructions, and quantitative analysis was performed based on the 2^−ΔΔCt^ method. The primer sequences of the target genes are detailed in the Table. S1.

### Cell transfection

RNASET2 siRNA was synthesized with the help of RiboBio (Guangzhou, China) and the sequence is shown in the Table. S1. Next, siRNA was transfected into A498 and 786-O cells following the manufacturer’s instructions method using Opti-MEM I and Lipofectamine 2000 (Invitrogen).

### Subcellular fractionation

Perform the procedure according to the manufacturer’s instructions, using the Cytoplasmic and Nuclear RNA Purification Kit (Thermo Scientific, USA).

### Wound healing assay

The A498 and 786-O cells were homogenously seeded in 6-well plates, treated accordingly and then the cells were scratched with 200 µL of pathogen-free pipette tip after full growth. The cells were then cultured in serum-free medium with photographs taken at 0 and 48 h after scratching and quantified using ImageJ software.

### Transwell assay

Approximately 2 × 10^4^ cells were suspended in 200 µL of serum-free medium and inoculated into the upper chamber of the Transwell, and 500 µL of medium containing 15–20% fetal bovine serum was added to the lower chamber. After 24 h of incubation, the cells in the lower chamber were fixed for 20 min and then stained for 15 min. Finally, photographs were taken under a microscope and counted using image J software.

### Matrigel tube formation assay

Culture supernatants of A498 and 786-O cells transfected with RNASET2 siRNA or negative control siRNA were collected and used to suspend HUVECs. The matrigel (Corning, NY, USA) is thawed overnight at 4 °C in the refrigerator, and the appropriate ratio and volume of matrigel mixture was added to each well of a 96-well culture plate and incubated at 37 °C for 30 min to solidify. Approximately 1.0 × 10^4^ HUVEC cells were inoculated into wells containing Matrigel coagulum. 2 h later, the formation of capillary-like structures was observed and photographed under a light microscope.

### Western blotting

After lysis of the treated cells in RIPA lysis buffer containing protease inhibitors, protein quantification by BCA (Cwbio, China) method and high temperature denaturation, the samples were separated electrophoresis on 10% SDS-PAGE gels and transferred to PVDF membranes (0.45 mm, Immobilon-P Transfer Membrane). Next, after being closed with 5% skimmed milk for 1–2 h at room temperature, they were incubated overnight at 4 °C with primary antibody. The details of the antibodies are shown in Table. S2. After performing 3–4 membrane washes using TBST, the protein bands were incubated with horseradish peroxidase-conjugated secondary antibodies at room temperature and again 3–4 washes using TBST. An enhanced chemiluminescence (ECL kit; FDbio, Hangzhou, China) detection system was used to visualize the results, analyzed by using ImageJ and GraphPad Prism 9.

### Transcriptome sequencing

As described in our previous studies, RNA from three pairs of kidney cancer cell species transfected with RNASET2 siRNAs (si-2) and si-NC were extracted and subsequently sent to Novogene Co., Ltd. (Beijing, China) for transcriptome sequencing.

### Immunohistochemistry

These concrete steps are in accordance with our previous study [20]. Fresh tissue samples were first fixed in 4% paraformaldehyde, dehydrated in ethanol, embedded in paraffin and sectioned. The slices were deparaffinized, rehydrated, soaked in sodium citrate and microwaved for antigen extraction. Sections were then blocked with 1% BSA and incubated with primary antibodies (anti-RNASET2, anti-Foxp3) overnight at 4 °C at concentrations of 1:100 and 1:200, respectively. Each section was incubated with the corresponding secondary antibody at 37 °C for 1 h. The slides were stained with diaminobenzidine and counterstained with hematoxylin. The images were collected using a Zeiss microscope and were analyzed using ImageJ software.

### Drug sensitivity analysis

To investigate the efficacy of RNASET2 expression on the treatment of some drugs used in cancer, the half-maximal inhibitory concentrations (IC50) of different samples were calculated using the cancer drug sensitivity genomics database and the “pRRophetic” package [21], as well as comparing the differences between high and low RNASET2 expression groups. The magnitude of the IC50 value is often inversely proportional to the drug sensitivity.

### Statistical analyses

Transcriptome sequencing data implemented in this study and RNA-Seq gene expression data obtained from TCGA and GEO databases were analyzed using R software (version 3.6.3). Wilcoxon signed rank test and Wilcoxon rank sum test were used for comparison between groups; ROC curves, Kaplan-Meier analysis and Cox analysis were used to identify the diagnostic and prognostic value of RNASET2 for ccRCC. Spearman correlation analysis was used to evaluate the association between mRNA expression of RNASET2 with tumor-infiltrating lymphocytes (TILs) and immune checkpoints, with *P* < 0.05 suggesting a statistically significant result.

## Results

### RNASET2 is upregulated in ccRCC

**Fig. 1:**
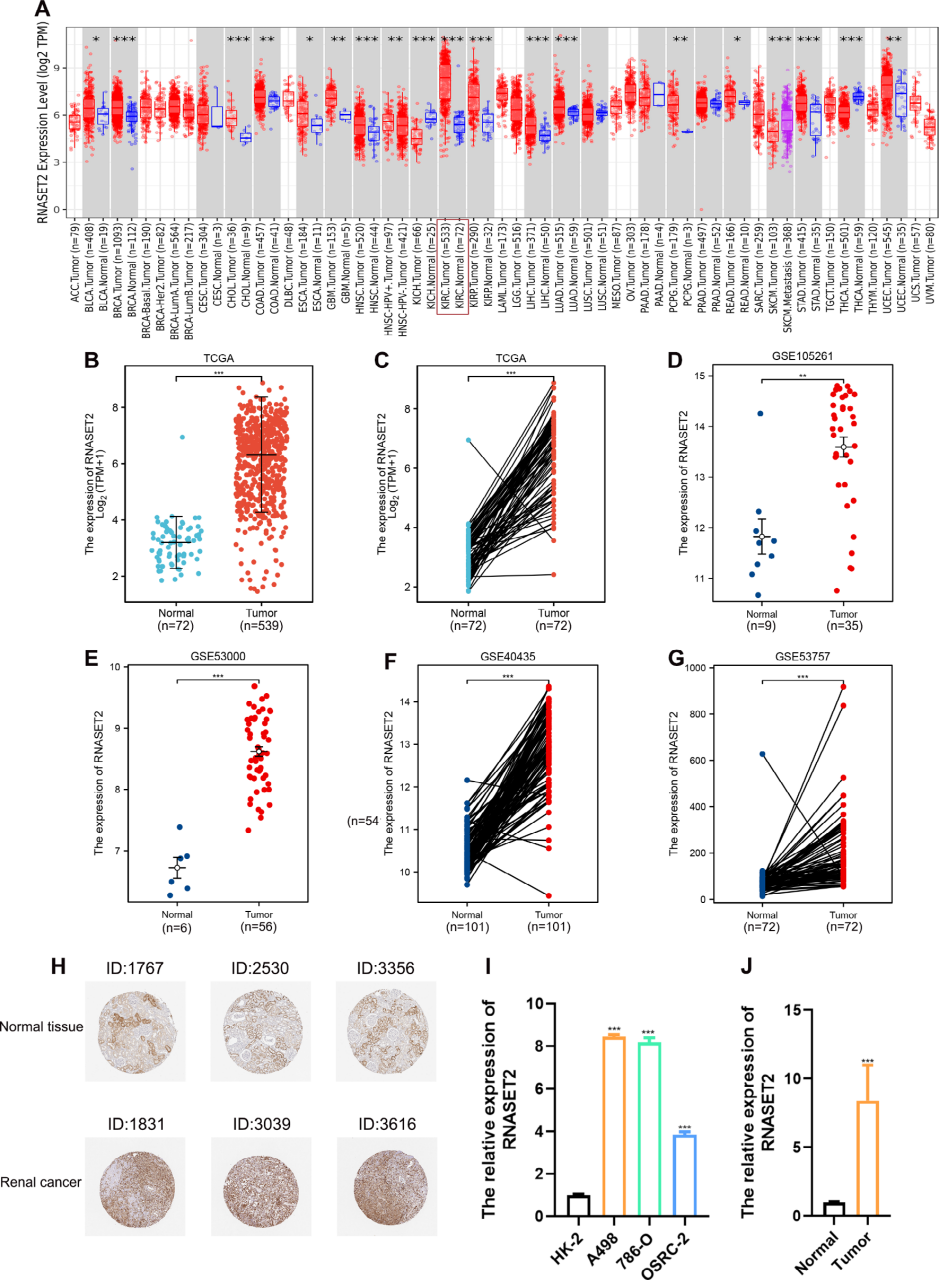
Upregulation of RNASET2 expression in ccRCC. (**A**) mRNA expression of RNASET2 in human cancer tissues. (**B, C**) mRNA expression of RNASET2 in unpaired and paired ccRCC tissues from the TCGA database. (**D-G**) mRNA expression of RNASET2 in GEO datasets (GSE105261, GSE53000, GSE40436, GSE53757). (**H**) Expression of RNASET2 in renal cancer tissues and normal kidney tissues revealed based on immunohistochemical staining. (**I, J**) qPCR validation of RNASET2 expression in renal cancer cell lines and ccRCC tissues collected at our centre. *p < 0.05; **p < 0.01; ***p < 0.001. RNASET2, Ribonuclease T2. ccRCC, clear cell renal cell carcinoma

First, we analyzed the expression of RNASET2 in human cancers and normal tissues based on the TCGA dataset. The results showed that RNASET2 was aberrantly highly expressed in a variety of cancers, including kidney, lung, breast and hepatocellular carcinomas (Fig. 1A). Next, we further analyzed RNASET2 mRNA expression in 539 ccRCC tissue samples and 72 paired samples in the TCGA database and found that RNASET2 was overexpressed in ccRCC tissues (Fig. 1, B to C). Consistently, this finding was also validated in the GEO datasets (GSE105261, GSE53000, GSE40436, GSE53757) (Fig. 1, D to G). Protein expression levels of RNASET2 in renal carcinoma were obtained from immunohistochemical staining data from the HPA database (antibody, HPA066509). Consistent with the upregulation of mRNA levels, RNASET2 protein expression was significantly upregulated in renal cancer tissues (patient numbers: 1831, 3039 and 3616) compared to normal tissues (patient numbers: 1767, 2530 and 3356) (Fig. 1H). Finally, the high expression of RNASET2 was verified using the renal cell carcinoma cell lines (A498, 786-O and OSRC-2) and HK-2, as well as 8 pairs of ccRCC tissues and adjacent normal tissues from our medical center (Fig. 1, I and J). Taken together, these results suggest that RNASET2 expression is highly regulated in ccRCC.

### Crosstalk between RNASET2 and clinicopathological features

Details of the clinicopathological characteristics of ccRCC patients obtained from the TCGA database and their relationship with RNASET2 expression are shown in Table 1. Meanwhile, we observed that T stage (*P* < 0.001) and M stage (*P* < 0.001) were significantly associated with RNASET2 mRNA expression. In addition, we also found that the risk of pathological stage and histological grade increased with the rise of RNASET2 mRNA expression in Fig. S1.

**Table 1:** Clinical Characteristics of ccRCC Patients and the Relationship with RNASET2 Expression

Characteristic	Low expression of RNASET2	High expression of RNASET2	P value
n	269	270	
Age, n (%)			1.000
<=60	134 (24.9%)	135 (25%)	
> 60	135 (25%)	135 (25%)	
Gender, n (%)			**< 0.001**
Female	145 (26.9%)	41 (7.6%)	
Male	124 (23%)	229 (42.5%)	
T stage, n (%)			**< 0.001**
T1	165 (30.6%)	113 (21%)	
T2	27 (5%)	44 (8.2%)	
T3	69 (12.8%)	110 (20.4%)	
T4	8 (1.5%)	3 (0.6%)	
N stage, n (%)			0.594
N0	130 (50.6%)	111 (43.2%)	
N1	7 (2.7%)	9 (3.5%)	
M stage, n (%)			**0.021**
M0	223 (44.1%)	205 (40.5%)	
M1	29 (5.7%)	49 (9.7%)	
Pathologic stage, n (%)			**< 0.001**
Stage I	164 (30.6%)	108 (20.1%)	
Stage II	26 (4.9%)	33 (6.2%)	
Stage III	46 (8.6%)	77 (14.4%)	
Stage IV	32 (6%)	50 (9.3%)	
Histologic grade, n (%)			**0.020**
G1	10 (1.9%)	4 (0.8%)	
G2	129 (24.3%)	106 (20%)	
G3	89 (16.8%)	118 (22.2%)	
G4	33 (6.2%)	42 (7.9%)	
OS event, n (%)			**< 0.001**
Alive	205 (38%)	161 (29.9%)	
Dead	64 (11.9%)	109 (20.2%)	
Age, mean ± SD	60.25 ± 11.8	61 ± 12.38	0.474

**Fig. 2:**
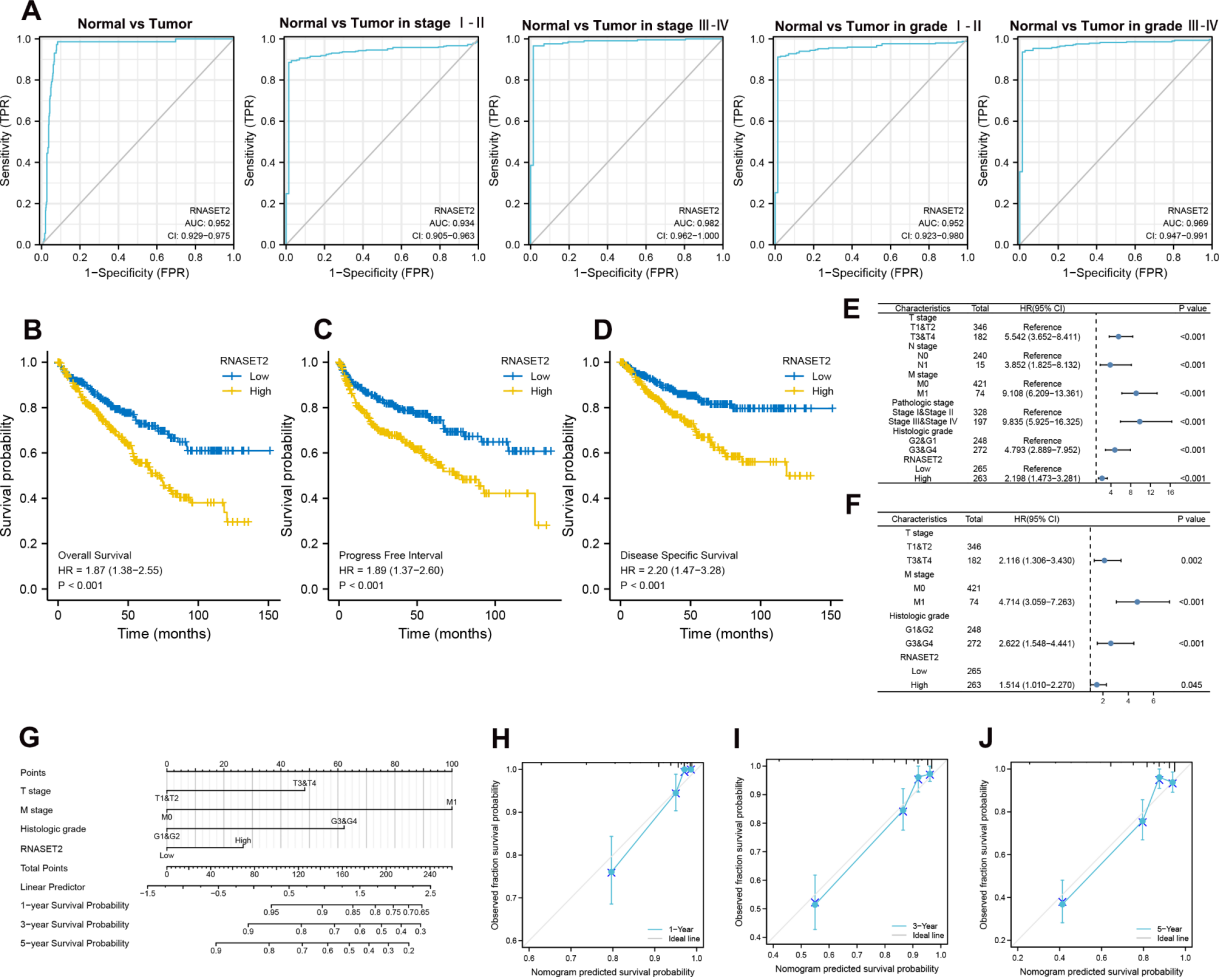
Clinical value of RNASET2 in ccRCC. (**A**) ROC curves revealing the diagnostic value of RNASET2 in ccRCC. (**B-D**) Kaplan-Meier curves showing the effect of RNASET2 mRNA expression on OS, DSS and PFI in ccRCC patients. (**E-F**) Cox regression forest plots evaluating independent prognostic factors for DSS in ccRCC patients. (**G-J**) Nomogram constructed based on independent prognostic factors and its calibration curve. RNASET2, Ribonuclease T2. ccRCC, clear cell renal cell carcinoma. OS, overall survival. DSS, disease-specific survival. PFI, progression-free interval

To further confirm the role of RNASET2 on the malignant biological behavior of ccRCC, we investigated the effect of RNASET2 expression on the clinical outcome of ccRCC patients. First, the receiver operating characteristic (ROC) curve was used to assess the diagnostic value of RNASET2 for kidney cancer. We identified that RNASET2 showed good diagnostic performance in different groups (area under the ROC curve was greater than 0.93 in all groups) (Fig. 2A). On the other hand, results revealed that upregulation of RNASET2 was associated with disease specific survival (DSS) (HR: 2.20; 1.47–3.28, *P* < 0.001), overall survival (OS) (HR: 1.87; 1.38–2.55, *P* < 0.001) and progression-free interval (PFI) (HR: 1.89; 1.37–2.60, *P* < 0.001) in ccRCC patients (Fig. 2, B and D). Subsequently, univariate and multivariate Cox regression analyses were performed to validate the independent prognostic value of RNASET2 for DSS in ccRCC patients; the results showed that T-stage, M-stage, histological grade and RNASET2 met the independent risk factor criteria for DSS (Fig. 2, E and F). Finally, we included the expression of T stage, M stage, histological grade and RNASET2 in ccRCC patients in a nomogram using the R package (“rms” & “survival”) to predict patient survival at 1, 3 and 5 years (Fig. 2G). The nomogram performed well in predicting the incidence of DSS in ccRCC, with a C-index of 0.826, and calibration plots predicting model performance showed good agreement (Fig. 2, H to J). In summary, high expression of RNASET2 is associated with poor outcome in ccRCC and may have high prognostic assessment and diagnostic potential.

### RNASET2 promotes in vitro RCC cell migration and in vitro angiogenesis

**Fig. 3:**
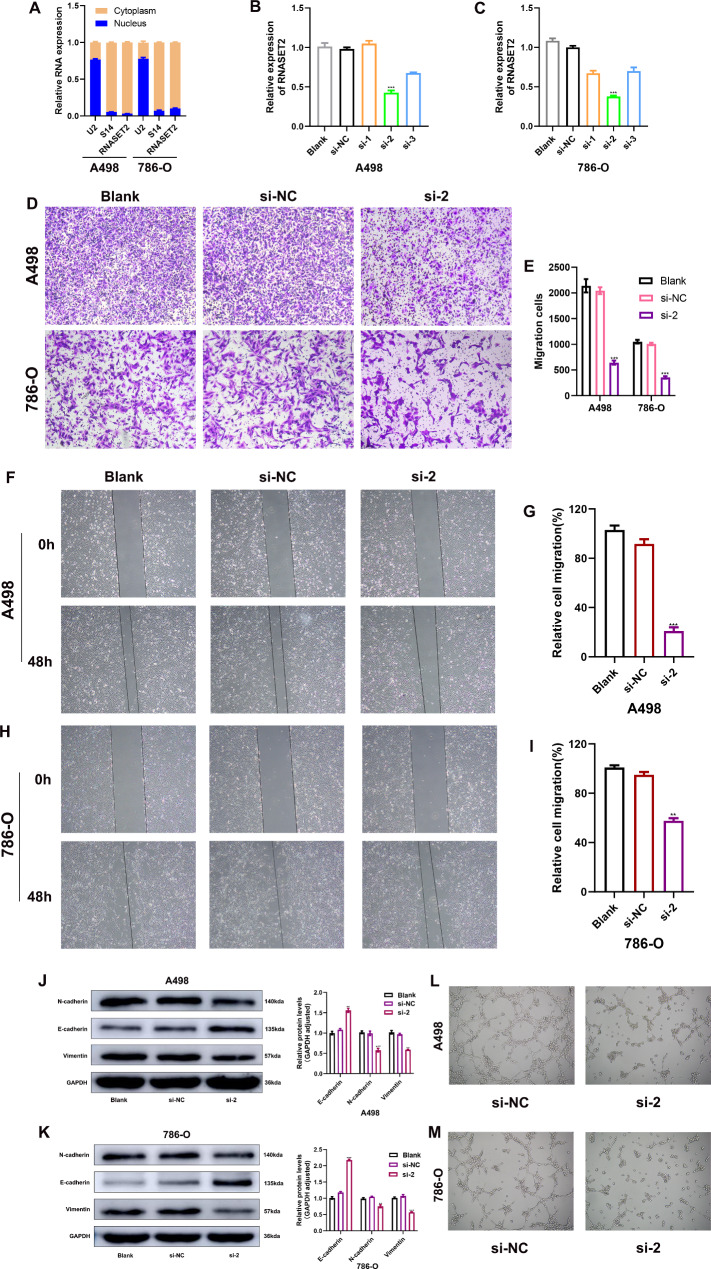
Inhibitory effects of RNASET2 knockdown on renal cancer cell migration and angiogenesis. (**A**) Subcellular localization of RNASET2 in renal cancer cell lines. (**B, C**) The knockdown efficiency of three RNASET2 siRNAs in kidney cancer cell lines A498 and 786-O. (**D, E**) The changes in migration ability of A498 and 786-O cells in Transwell assay after knockdown of RNASET2. Scale bars, 50 μm. (**F-I**) The wound healing assay of A498 and 786-O cells after silencing RNASET2 expression. Scale bars, 200 μm. (**J, K**) Changes in EMT process-related proteins in renal cancer cells after RNASET2 knockdown. (**L, M**) Representative matrigel tube formation. Scale bars, 100 μm. (**L**) Matrigel tube formation of HUVECs cultured with conditioned medium from A498 negative control (si-NC)and knock-down (si-2) groups. (**M**) Matrigel tube formation of HUVECs cultured with conditioned medium from 786-O negative control(si-NC) and knockdown (si-2)groups. **, p < 0.01, ***, p < 0.001. RNASET2, Ribonuclease T2. EMT, epithelial–mesenchymal transition. NC, negative control

First, with the help of subcellular fractionation experiments, we determined that RNASET2 expression was mainly located in the cytoplasm of kidney cancer cells (A498, 786-O) (Fig. 3A). Next, we treated A498 and 786-O cells with siRNA to knock down RNASET2 expression in the kidney cancer cell lines and validate its knockdown efficiency, accompanied by subsequent functional assays (Fig. 3, B and C). Interestingly, Transwell analysis and Wound healing results showed that silencing RNASET2 significantly inhibited the migration of A498 and 786-O cells in vitro (Fig. 3, D and I). In parallel, we analyzed the effect of knockdown of RNASET2 on the epithelial–mesenchymal transition (EMT) process by Western Blotting. The results showed that E-Cadherin expression was significantly up-regulated and N-Cadherin and Vimentin expression was remarkably inhibited after treatment of A498 and 786-O cells with RNASET2 siRNA (Fig. 3J and K). This implies that RNASET2 can promote the EMT process in kidney cancer cells to some extent, which is also consistent with the results of cell migration assays. Given the close association of RNASET2 with angiogenesis in other cancers, we explored its impact on this malignant phenotype of ccRCC [14]. However, inconsistent with previous findings, the angiogenic capacity of HUVECs was significantly reduced after treatment with culture medium supernatant from renal cancer cells (A498, 786-O) following RNASET2 siRNA intervention relative to the si-NC group (Fig. 3, L and M). This evidence suggests that RNASET2 may be involved in promoting the process of angiogenesis in ccRCC.

### Sequencing analysis after knockdown of RNASET2 in RCC cells

**Fig. 4:**
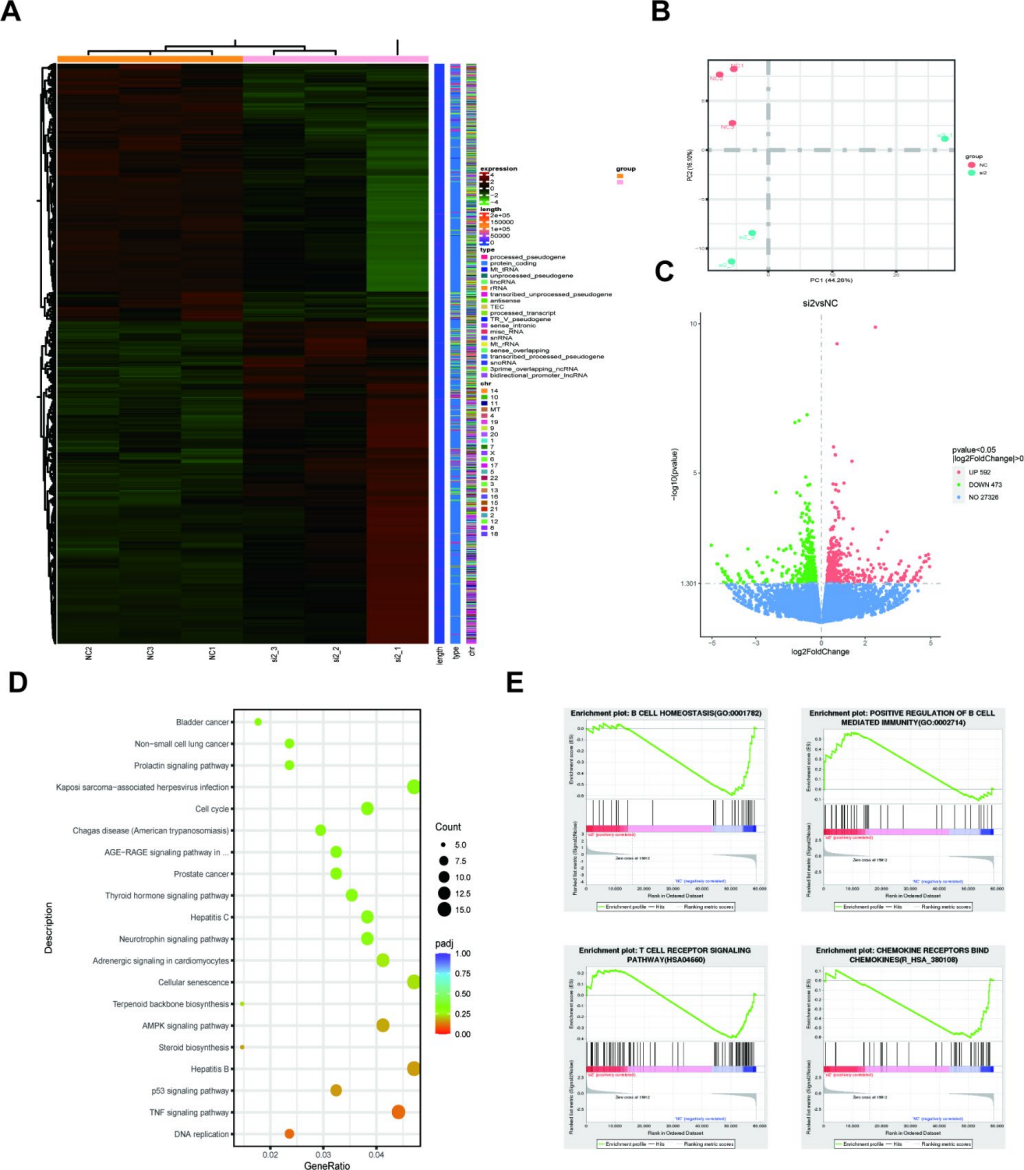
Analysis of sequencing results after RNASET2 knockdown. (**A**) Heat map of gene expression after RNASET2 knockdown. (**B**) PCA plot showing the altered signature after RNASET2 knockdown. (**C**) Volcano map showing up- and down-regulated genes after RNASET2 knockdown. (**D, E**) Plots of KEGG enrichment analysis (www.kegg.jp/kegg/kegg1.html) and GSEA results for differential genes following RNASET2 knockdown. RNASET2, Ribonuclease T2

Total RNA from 3 pairs of si-RNASET2 and si-NC treated A498 cells was subjected to transcriptome sequencing analysis to systematically elucidate the biological basis of RNASET2 in RCC. Differentially expressed genes in the si-RNASET2 and NC groups are shown in Fig. 4A, and gene expression within the groups showed good concordance, a trend also confirmed by Principal Component Analysis (PCA) (Fig. 4B). In addition, we constructed a volcano plot to visualize the distribution of differentially expressed genes in the two groups (Fig. 4C). There were 592 genes significantly up-regulated and 473 genes significantly down-regulated in the si-RNASET2 group compared to the si-NC group.

Using differentially expressed genes in both groups, we explored the potential biological pathways of RNASET2 in ccRCC. Kyoto Encyclopedia of Genes and Genome (KEGG) analyses suggested that the AMPK signaling pathway, the P53 signaling pathway, and the TNF signaling pathway were significantly enriched [22] (Fig. 4D). Besides, genes related to the VEGF signaling pathway were also altered accordingly after RNASET2 was knocked down [22] (Fig. S2), which further corroborated the results of tube formation assays. Furthermore, gene set enrichment analysis (GSEA) showed that knockdown of RNASET2 resulted in up- or down-regulation of genes constituting the B-cell homeostasis, B-cell immunity, chemokine and chemokine receptor binding, and T-cell receptor signaling pathways (Fig. 4E). This evidence supports the possibility that RNASET2 plays a regulatory role in the immune microenvironment of ccRCC.

### Association of RNASET2 with immune characteristics

**Fig. 5:**
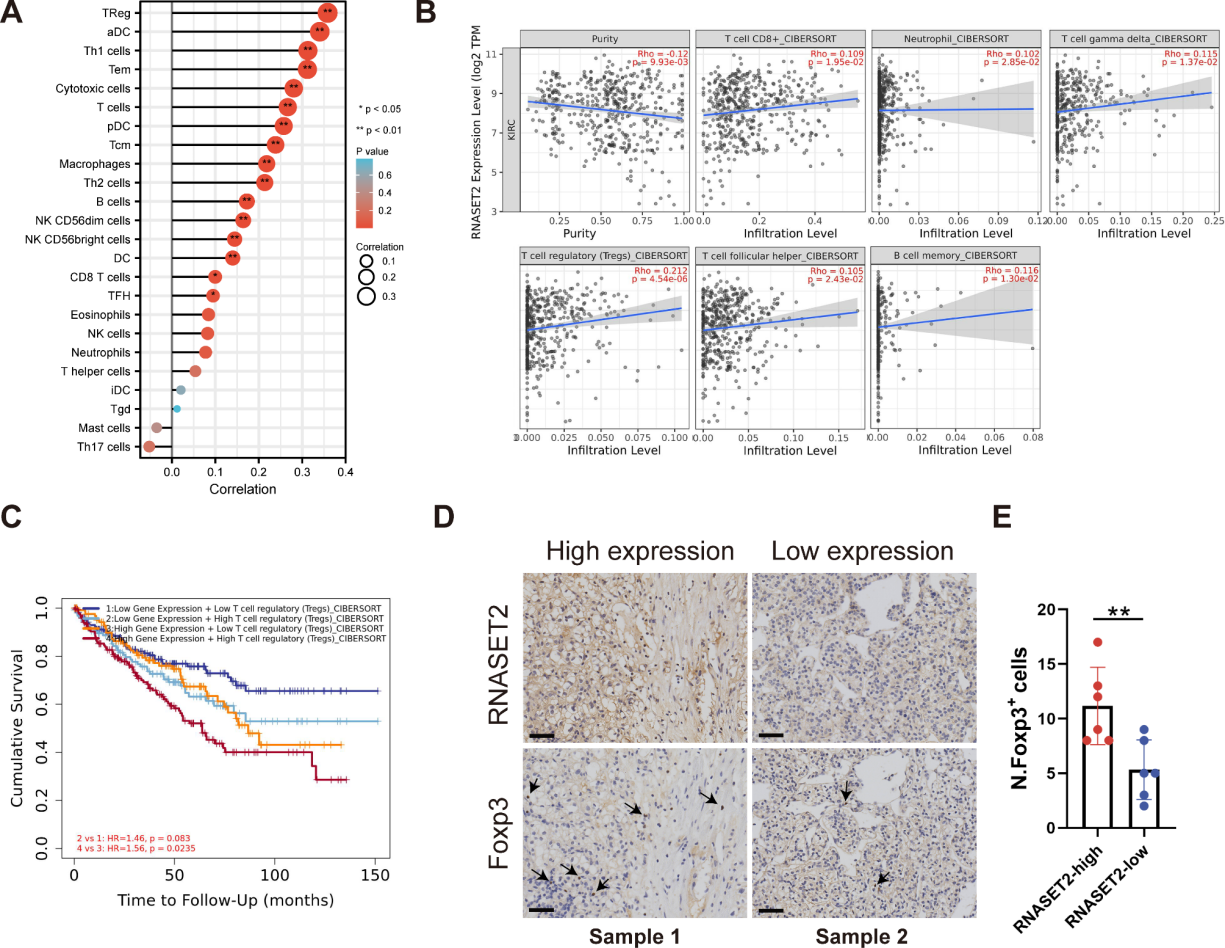
RNASET2 is associated with immune lymphocytes in ccRCC. (**A, B**) Correlation of RNASET2 with immune lymphocytes revealed based on ssGSEA and CIBERSORT algorithms. (**C**) Effect of Regulatory T cell enrichment status combined with RNASET2 expression on prognosis of ccRCC patients. (**D-E**) Immunohistochemical staining demonstrated the relationship between RNASET2 expression levels and the enrichment of regulatory T cells. Scale bars, 50 μm. *, p < 0.05, **, p < 0.01. RNASET2, Ribonuclease T2. ccRCC, clear cell renal cell carcinoma

Given that RNASET2 may be involved in the regulation of the immune microenvironment in ccRCC, we then analyzed the crosstalk between it and immune features. First, with the help of the ssGSEA and CIBERSORT algorithms, we performed an analysis of the correlation between RNASET2 and TILs (Fig. 5A, B). The results showed that RNASET2 was positively correlated with regulatory T cells (Tregs). Notably, enrichment of enrichment of Tregs was associated with poor outcome in ccRCC patients (Fig. 5C). To further validate the correlation between RNASET2 and Tregs, we collected 12 ccRCC tumor samples for immunohistochemical staining analysis, and Foxp3 was used to label Tregs. The results showed that the number of Tregs infiltrated was more in ccRCC samples with high RNASET2 expression relative to low expression samples (Fig. 5, D and E).

**Fig. 6:**
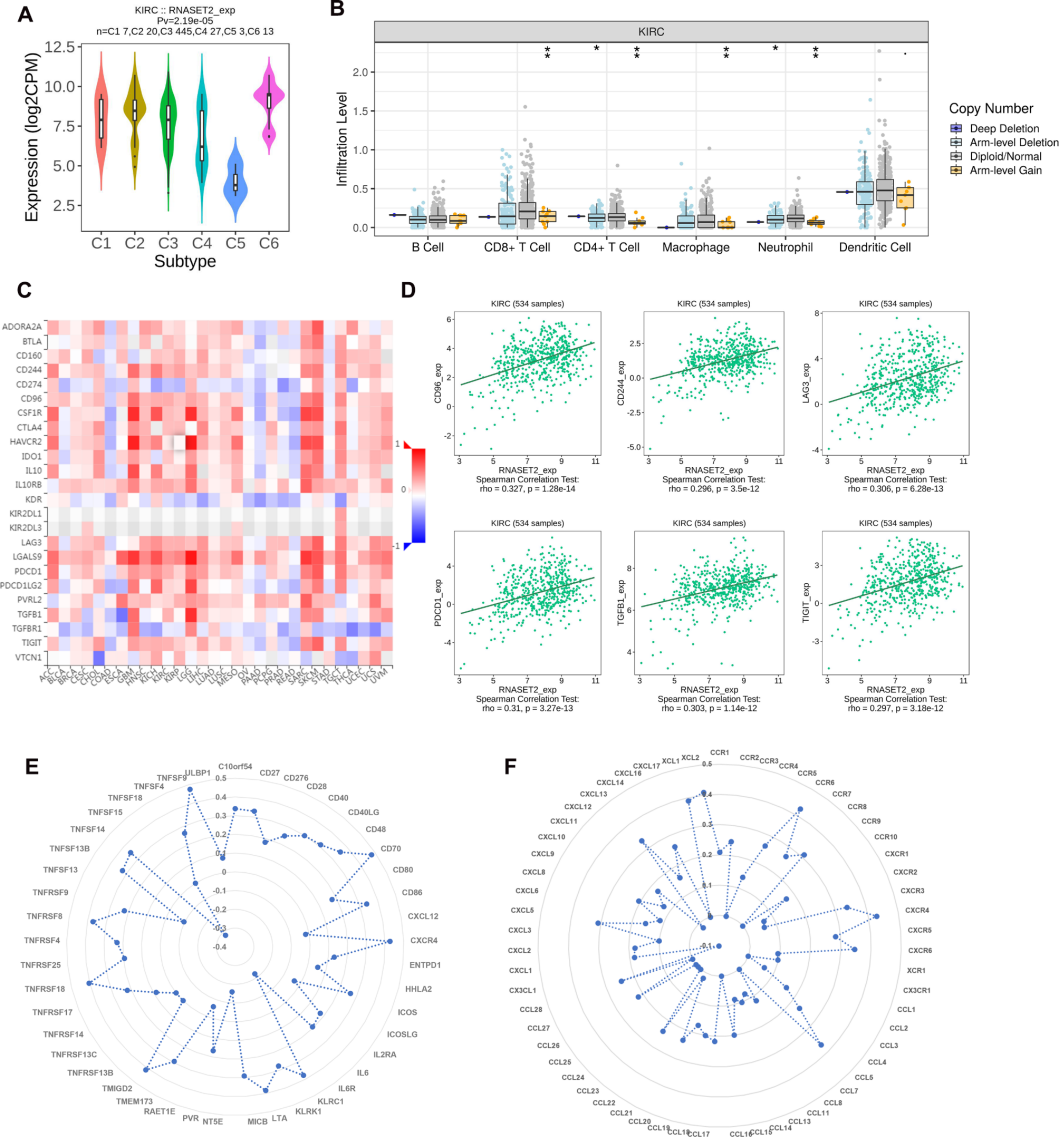
Association of RNASET2 with cancer subtypes of ccRCC, immune checkpoint genes and chemokines/receptors. (**A**) Relationship between RNASET2 mRNA expression and immune subtypes in ccRCC. (**B**) Changes in immune subpopulation infiltration at different copy number status of RNASET2. (**C, D**) Correlation analysis of RNASET2 mRNA expression with immunosuppressive checkpoints in ccRCC. (**E**) Correlation analysis of RNASET2 mRNA expression with immunostimulator in ccRCC. (**F**) Correlation analysis of RNASET2 with chemokines and receptors in ccRCC. *, p < 0.05, **, p < 0.01. RNASET2, Ribonuclease T2. ccRCC, clear cell renal cell carcinoma

Furthermore, using the TISIDB database, we analyzed the relationship between RNASET2 and the immune subtypes of ccRCC. As shown in Fig. 6A, RNASET2 was significantly highly expressed in type C2 (INF-γ dominant) and type C3 (inflammatory), while it was expressed least in type C5 (immunologically quiet). This implies that RNASET2 expression is directly related to the immune microenvironment of ccRCC. Genes with somatic copy number alterations (SCNAs) are considered hallmarks of human cancer development and progression and may influence immunotherapy response [23, 24]. Based on the SCNA module of the TIMER2.0 database, we explored the potential link between the copy number alterations of RNASET2 and infiltration levels of TILs in the tumor microenvironment of ccRCC. In Fig. 6B, arm-level gains in RNASET2 copy number were associated with reduced abundances of CD8 + cells, CD4 + cells, macrophages and neutrophils compared to the diploid/normal state. These results suggest that the copy number changes of RNASET2 in ccRCC may be one of the factors that regulate the immune microenvironment.

Immune checkpoints are directly linked to a state of immunosuppression in the tumor microenvironment, making immune checkpoint inhibitor-based systemic therapies a prime option for patients with advanced ccRCC [4]. Here, we found that mRNA expression of RNASET2 was closely associated with immunosuppressive checkpoints in a variety of human cancer tissues (Fig. 6C). In ccRCC, mRNA levels of RNASET2 were positively correlated with the expression levels of CD96, CD244, LAG3, PDCD1, TGFβ1 and TIGIT (Fig. 6D). Besides, the interaction of RNASET2 with immunostimulator in is shown in Fig. 6E. On the other hand, considering that GSEA suggests that knockdown of RNASET2 may be involved in regulating the binding process of chemokines to receptors, we further visualized the relationship between RNASET and the expression levels of chemokines and receptors in ccRCC. As shown in Fig. 6F, the mRNA expression of RNASET2 was positively correlated with CXCR4, CCL7, CCR6, etc. Notably, these molecules have been found to promote the malignant phenotype of ccRCC in previous studies [25–27].

### Analysis of RNASET2 gene mutations and drug sensitivity in ccRCC

**Fig. 7:**
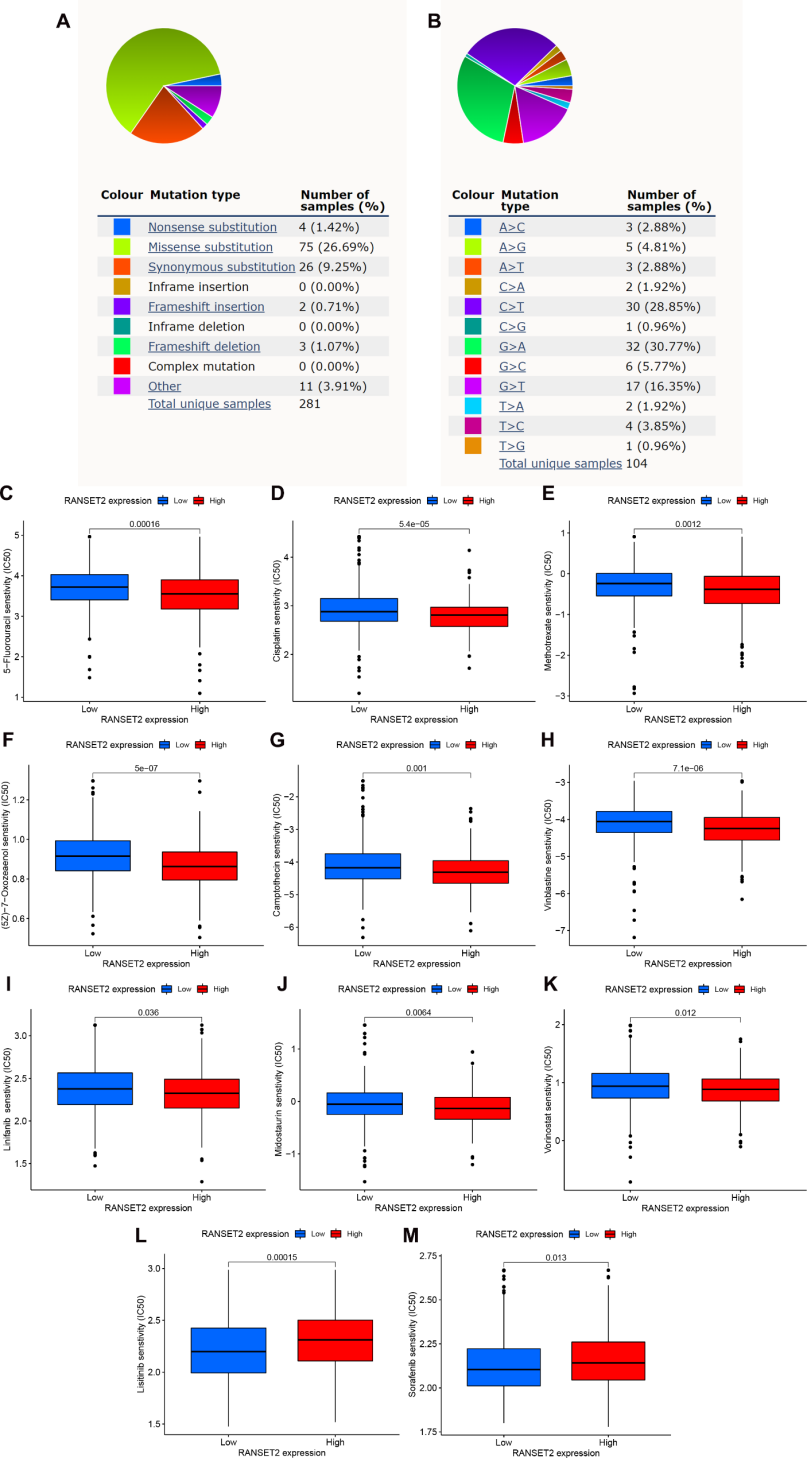
Mutation and drug sensitivity analysis of RNASET2 in ccRCC patients. (**A, B**) Distribution of different types of RNASET2 mutations in cancer. (**C-M**) Relationship between drug sensitivity and RNASET2 expression. RNASET2, Ribonuclease T2. ccRCC, clear cell renal cell carcinoma

Frist, the mutation types of RNASET2 were evaluated in the COSMIC database. As shown in Fig. 7A, the missense substitutions occurred in about 26.69% of the samples, synonymous substitutions in 9.25%, and nonsense substitutions in 1.42% of the samples. Substitution mutations included G > A (30.77%), C > T (28.85%), G > T (16.35%) and G > C (5.77%) (Fig. 7B). In addition, results based on the GSDC database showed that RNASET2 expression could predict drug sensitivity to some extent. The results showed that patients with high RNASET2 expression were associated with lower IC50 values for some chemotherapeutic agents, such as 5-Fluorouracil, cisplatin, methotrexate, (5Z)-7Oxozeaenol and camptothecin, suggesting that patients with high RNASET2 expression were more sensitive to these drugs (Fig. 7, C to G). Similarly, high expression of RNASET2 in some targeted therapeutic agents increases the sensitivity of patients to the drug, such as vinblastine, linifanib, midostaurin, and vorinostat (Fig. 7, H to K). Interestingly, increased expression of RANSET2 decreases the sensitivity of patients to lisitinib and sorafenib (Fig. 7, L and M). Taken together, the detection of RNASET2 expression levels may be helpful in guiding the clinical dosing of ccRCC patients.

## Discussion

**Fig. 8:**
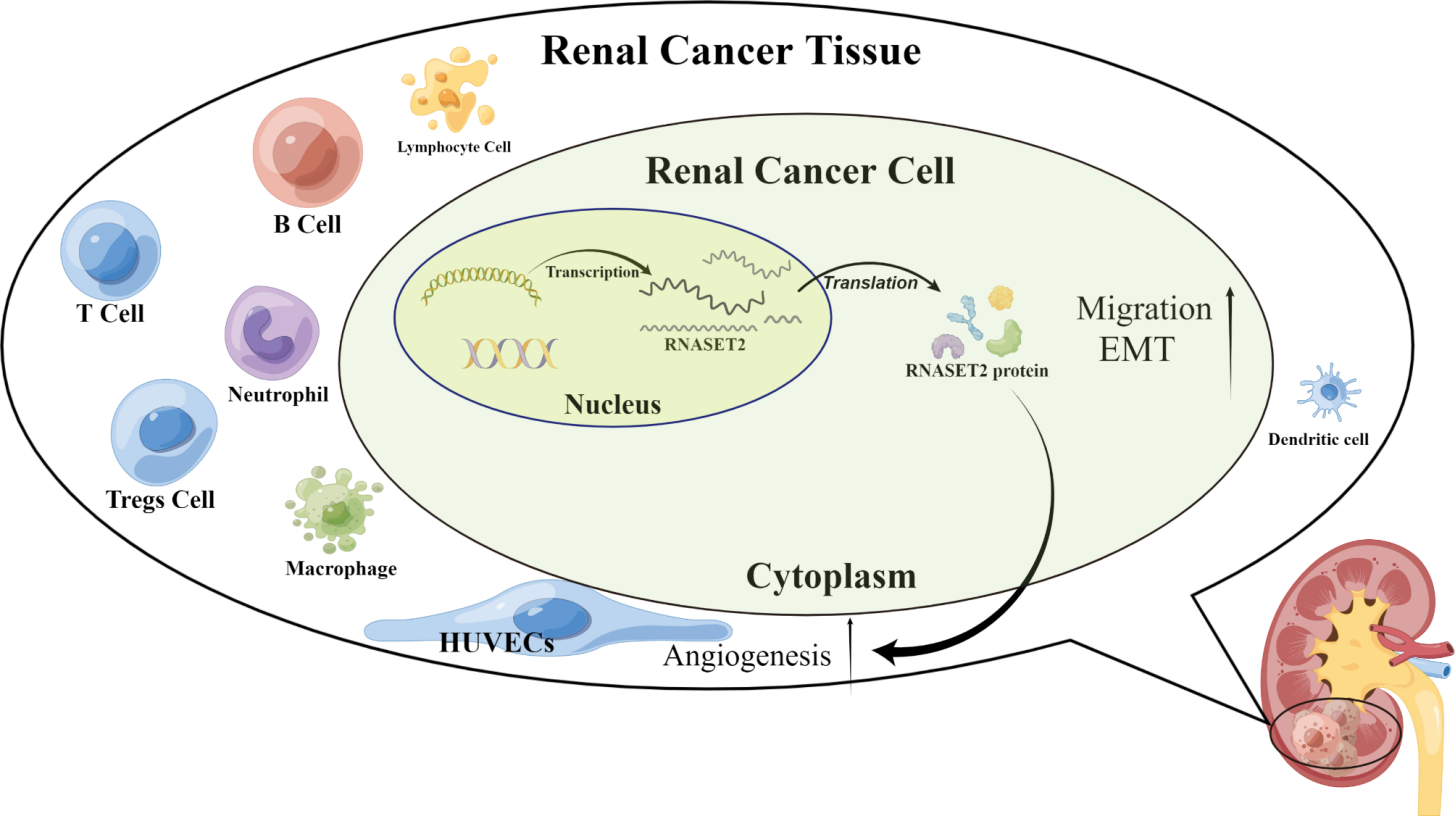
Schematic representation of the mechanism of RNASET2 in RCC cells

The treatment of advanced ccRCC has undoubtedly evolved significantly in recent years as a better understanding of the molecular composition of the disease has emerged [28]. Anti-angiogenic therapy and immunotherapy, the cornerstones of systemic therapy for advanced ccRCC, have improved the clinical outcome of many patients. However, durable and precise anti-tumor efficacy remains a goal pursued by urologists and researchers. Here, through bioinformatics and in vitro experimental validation, we identified aberrant high expression of RNASET2 in ccRCC and characterized its diagnostic and prognostic value. In addition, the relationship of RNASET2 with the angiogenic process and immune microenvironment in ccRCC has been preliminarily elucidated, as detailed in Fig. 8.

RNASET2 is widely present in the genomes of animals, plants, bacteria and viruses [29]. In human cancers, previous evidence suggests that it is associated with tumor suppressors. For example, RNASET2 expression is down-regulated in various ovarian tumor and cancer cell lines, and its introduction into cells exerts tumor suppressive effects both in vivo and in vitro [14, 30]. At the same time, this anti-tumor effect was found to be unaffected by double point mutations in the catalytic site of the targeted putative ribonuclease, as inactivation of the T2 enzyme by mutation or denaturation did not block the tested anticancer effect [14, 30]. However, in gastric adenocarcinoma, RNASET2 expression was shown to have no significant correlation with angiogenesis, lymph node metastasis or patient prognosis, despite being significantly downregulated relative to normal tissue [11]. In the present study, we found that RNASET2 was significantly highly expressed in ccRCC tissues and correlated with poor prognosis of patients. Furthermore, silencing of RNASET2 inhibited migration of renal cancer cells and angiogenesis in the tumor microenvironment. Indeed, this finding contradicts our conventional knowledge of RNASET2 in human cancers and the reasons for this paradox are still unknown. What is certain, however, is that this evidence goes some way to improving our understanding of RNASET2 and ccRCC.

ccRCC is known to be a highly immuno-infiltrative tumor with an immune microenvironment consisting of TILs, cytokines secreted by TILs, chemokines and immune checkpoints [31]. Here, through transcriptome sequencing analysis of si-RNASET2(si-2) and si-NC treated renal cancer cells and subsequent exploration of biological mechanisms, we found that RNASET2 may be involved in the regulation and remodeling of the immune microenvironment in renal cancer to some extent. It is certain that TILs occupy a central role in the immune microenvironment of ccRCC, and different types and different infiltration abundance of TILs can directly influence the anti-tumor immune response of the body. For instance, Tregs are one of the major immunosuppressive cell types in malignancies, and their abundance in the tumor microenvironment is closely linked to disease progression [32]. Interestingly, we found a significant positive correlation between RNASET2 and Tregs infiltration abundance by bioinformatics analysis and immunohistochemical staining analysis, highlighting its immunosuppressive properties and malignant phenotype. Furthermore, RNASET2 expression was found to be closely associated with the immune subtype of ccRCC, and the copy number of RNASET2 was suggested to possibly influence the abundance of TILs, further corroborating the immune properties of RNASET2 in ccRCC.

Immune checkpoints are also a key component of the tumor immune microenvironment, and blockade of some immunosuppressive checkpoints in particular can significantly enhance the body’s anti-tumor immune response [33]. Intriguingly, our results show that RNASET2 is significantly positively correlated with CD96, CD244, LAG3, PDCD1, TGFβ1 and TIGIT, reaffirming the immunosuppressive properties of RNASET2 in ccRCC. Since LAG3, PDCD1 and TIGIT have long been identified as key immune checkpoints in ccRCC, their blockade has demonstrated satisfactory anti-tumor efficacy in preclinical and clinical studies [34–36]. In addition, CD96, CD244 have been identified as new checkpoint receptor targets for cancer immunotherapy [37, 38], and TGFβ1 has been found to play a key role in the EMT process in ccRCC [39]. Advanced ccRCC often causes systemic inflammation and the cancer cells reshape the immune landscape by secreting cytokines or chemokines [40]. Evidence suggests that enhanced expression of CCL7 can promote cell growth and metastasis in renal cancer [26]. Similarly, silencing of CXCR4 impedes cancer progression and increases cisplatin sensitivity in ccRCC [41]. The CCL20-CCR6 axis, on the other hand, promotes cancer progression by enhancing the migration and proliferation of multiple human cancer cells and indirectly reshapes the tumor microenvironment by regulating TILs [42]. Interestingly, our data show that RNASET2 is closely associated with the upregulation of the expression of these molecules in ccRCC. This implies that the malignant phenotype of RNASET2 in ccRCC may be partially dependent on the interaction of these chemokines and receptors.

It is well known that spontaneous genetic mutations accumulate in somatic cells over the course of a person’s lifetime. Although most mutations have no apparent effect on the individual, some can alter critical cellular functions. For instance, the gradual accumulation of mutations over a lifetime may lead to cancer and cause aging [43]. There are many mutated genes have been reported to be associated with clear cell renal cell carcinoma, including von Hippel Lindau(VHL) [44], Polybromo 1 (PBRM1) [45], BRCA associated protein 1 (BAP1) [46] and SET domain containing 2 (SETD2) [47]. Deletion or mutation of the VHL gene is commonly considered to be an exclusive initiation step in the development of ccRCC [44]. Therefore, studying mutations in the ccRCC gene to determine their impact on the prognosis and treatment of patients with kidney cancer could provide additional benefits to patients. In this study, missense substitution was the most common type of mutation in RNASET2 among 281 cancer samples, present in 75 (26.69%); synonymous substitution was present in 26 cases (9.25%) (Fig. 7A). Substitution mutations included G > A (30.77%), C > T (28.85%), G > T (16.35%) and G > C (5.77%) (Fig. 7B). Overall, RNASET2 mutations are common in ccRCC. However, whether these mutations affect the progression of ccRCC remains unclear and needs to be explored in depth by subsequent studies.

It is well known that patients with metastatic ccRCC are usually deprived of surgery and have to be treated medically to prolong their survival. 5-Fluorouracil, used as a first-line treatment for metastatic renal cell carcinoma, in combination with interleukin-2 and interferon-alpha, has high responsiveness and is therapeutically effective [48–50]. As a class II histone deacetylase (HDAC) inhibitor vorinostat in combination with the vascular endothelial growth factor (VEGF) inhibitor bevacizumab has been relatively well tolerated and clinical efficacy in the treatment of patients with metastatic ccRCC [51]. However, the sensitivity of these drugs varies widely among individuals, and biomarkers predicting drug sensitivity are urgently needed to individualize treatment. Here, we found that patients with high RNASET2 expression were more sensitive to most of the drugs, and insensitive only to lisitinib and sorafenib. These results suggest that RNASET2 upregulation not only predicts a poor prognosis for ccRCC, but its expression level might also serve as a potent biomarker to guide the clinical treatment of patients with metastatic ccRCC.

Despite the systematic analysis of the role of RNASET2 in ccRCC in this study, there are still some limitations that need to be raised. Firstly, the clinicopathological information of ccRCC patients in this study was obtained from public databases and lacked validation from the dataset at the center of this study. Secondly, the effect of RNASET2 on the malignant behavior of renal cancer cells has only been explored in in vitro experiments and evidence from in vivo experiments needs to be refined in subsequent experiments. In the future, we will fill these deficiencies and refine relevant experiments. Overall, however, we present for the first time the value of RNASET2 for cell migration, angiogenesis and analysis of its immunosuppressive properties in ccRCC. These findings help to refine the molecular landscape of ccRCC and may provide new ideas for the treatment of advanced ccRCC.

## Conclusion

Our evidence suggests that RNASET2 expression is upregulated in ccRCC tissues and cell lines and is associated with malignant behavior of cancer cells and poor prognosis of patients. Mechanistically, its pro-cancer effects may depend on the promotion of renal cancer cell migration, angiogenesis, and remodeling of the immune microenvironment of RNASET2.

### Electronic supplementary material

Below is the link to the electronic supplementary material.

Supplementary Material 1

Supplementary Material 2

Supplementary Material 3

## Data Availability

Both RNA-seq and clinicopathological data for ccRCC patients are available from the database (https://portal.gdc.cancer.gov/) and the GEO database (https://www.ncbi.nlm.nih.gov/gds/; GPL10558, GPL6244, GPL13112, GPL570).

## Abbreviations

ccRCC: Clear Cell Renal Cell Carcinoma
RNASET2: Ribonuclease T2
TCGA: The Cancer Genome Atlas
GEO: Gene Expression Omnibus
qPCR: Quantitative polymerase chain reaction
GDSC: Genomics of Drug Sensitivity in Cancer
mRCC: Metastatic Renal Cell Carcinoma
HPA: Human Protein Atlas
COSMIC: Catalogue of Somatic Mutations in Cancer
HK-2: Human renal tubular epithelial cells
DMEM-H: Dulbecco’s modified Eagle medium-high glucose
HUVECs: The Human Umbilical Vein Endothelial Cells
ROC: Receiver Operating Characteristic
DSS: Disease Specific Survival
OS: Overall Survival
PFI: Progression-Free Interval
EMT: Epithelial–Mesenchymal Transition
PCA: Principal Component Analysis
ssGSEA: Single Sample Gene Set Enrichment algorithm
GO: Gene Ontology
KEGG: Kyoto Encyclopedia of Genes and Genomes
TILs: Tumor-Infiltrating Lymphocytes
SCNAs: Somatic Copy Number Alterations
TGF-β: Transforming Growth Factor-β
VEGF: Vascular Endothelial Growth Factor
