# Supplementary material for: Systematic analysis of RNASET2 gene as a potential prognostic and immunological biomarker in clear cell renal cell carcinoma
Source: BMC Cancer. 2023 Sep 7;23:837. doi: 10.1186/s12885-023-11356-6 (PMC10483861; doi:10.1186/s12885-023-11356-6)
Supplement: Supplementary file 1 — Supplementary Material 1 [file 12885_2023_11356_MOESM1_ESM.docx]

**Additional file 1 Table S1**. The primers for RT-qPCR and the sequence of RNASET2 siRNA

| **Gene** | **Forward or Reverse** | **Primer sequence** |
| --- | --- | --- |
| RNASET2 | Forward | 5’-AATAGATCGTGGCCCTTCA-3’ |
|  | Reverse | 5’-TTCCCACTCATGCTTCCA-3’ |
| β-actin | Forward | 5’-TCTCCCAAGTCCACACAGG-3’ |
|  | Reverse | 5’-GGCACGAAGGCTCATCA-3’ |
| U2 | Forward | 5’-CATCGCTTCTCGGCCTTTTG-3’ |
|  | Reverse | 5’-TGGAGGTACTGCAATACCAGG-3’ |
| S14 | Forward | 5’-GGCAGACCGAGATGAATCCTC-3’ |
|  | Reverse | 5’-CAGGTCCAGGGGTCTTGGTCC-3’ |
| hs-RNASET2-si-NC | Forward | 5’-UUCUCCGAACGUGUCACGUTT-3’ |
| hs-RNASET2-si-NC | Reverse | 5’-ACGUGACACGUUCGGAGAATT-3’ |
| hs-RNASET2-si-1 | Forward | 5’-GAUGAGGAAGUACAGACAATT-3’ |
| hs-RNASET2-si-1 | Reverse | 5’-UUGUCUGUACUUCCUCAUCTT-3’ |
| hs-RNASET2-si-2 | Forward | 5’-GCAGAAGCCUGGAACUCUATT-3’ |
| hs-RNASET2-si-2 | Reverse | 5’-UAGAGUUCCAGGCUUCUGCTT-3’ |
| hs-RNASET2-si-3 | Forward | 5’-CAGAGUAUAUGGAGUGAUATT-3’ |
| hs-RNASET2-si-3 | Reverse | 5’-UAUCACUCCAUAUACUCUGTT-3’ |

**Additional file 1 Table S2**. Antibodies used for WB

| **Antibody** | **Description** | **Supplier** | **City** | **Country** |
| --- | --- | --- | --- | --- |
| Anti-E-Cadherin | Rabbit | CST(3195T) | Boston | USA |
| Anti-N-Cadherin | Rabbit | CST(13116T) | Boston | USA |
| Anti-Vimentin | Rabbit | CST(5741T) | Boston | USA |
| Anti-GAPDH | Mouse | ZSGB-BIO(TA-08) | Beijing | China |
| Anti-RNASET2 | Rabbit | Proteintech(13753-1-AP) | Wuhan | China |
| Anti-Foxp3 | Rabbit | Servicebio(GB11093) | Wuhan | China |
