# Supplementary material for: Systematic analysis of RNASET2 gene as a potential prognostic and immunological biomarker in clear cell renal cell carcinoma
Source: BMC Cancer. 2023 Sep 7;23:837. doi: 10.1186/s12885-023-11356-6 (PMC10483861; doi:10.1186/s12885-023-11356-6)
Supplement: Supplementary file 2 — Supplementary Material 2 [file 12885_2023_11356_MOESM2_ESM.docx]

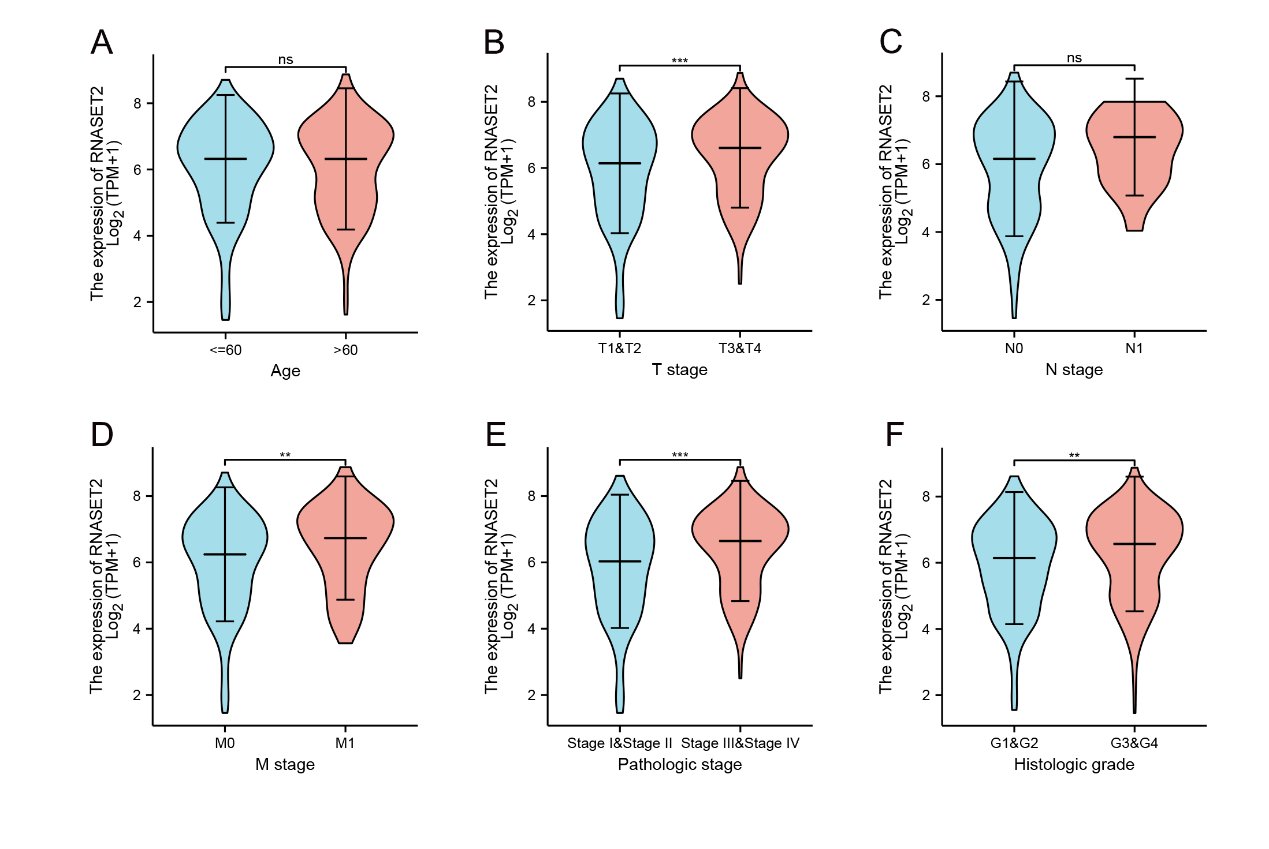


**Fig. S1.** Relationship between mRNA expression levels of RNASET2 and clinicopathological features. mRNA expression of RNASET2 did not correlate significantly with (A) patient age and (C) lymph node metastasis, whereas high expression was observed in patients with (B) T3-T4 stage, (D) distant metastases, (E) high pathological stage, and (F) high histological grade. **, p < 0.01, ***, p < 0.001. RNASET2, Ribonuclease T2. ccRCC, clear cell renal cell carcinoma.


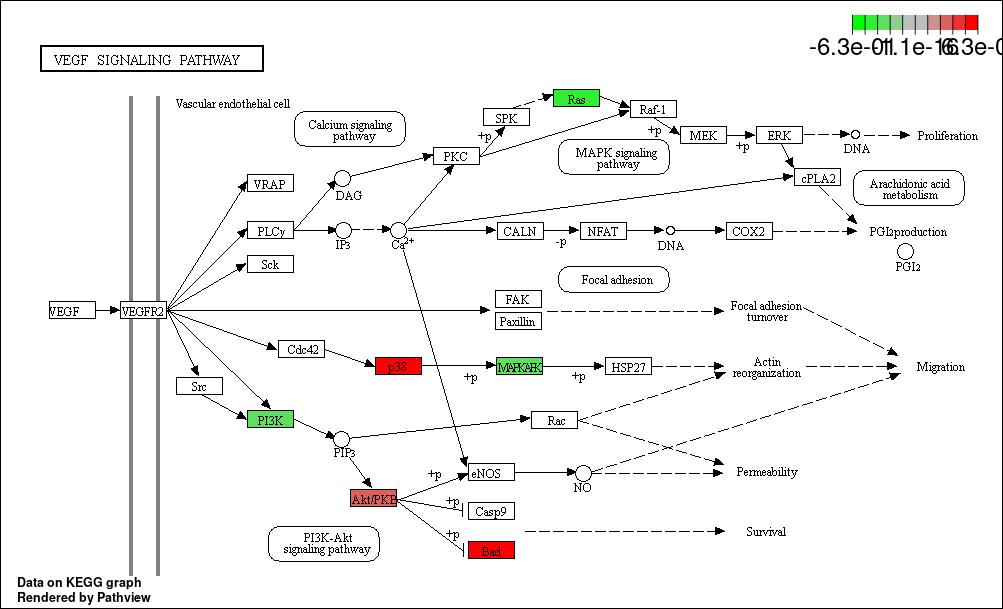


**Fig. S2.** The VEGF signaling pathway (www.kegg.jp/kegg/kegg1.html).
