# Supplementary figures and images for: Systematic analysis of RNASET2 gene as a potential prognostic and immunological biomarker in clear cell renal cell carcinoma
Source: BMC Cancer. 2023 Sep 7;23:837. doi: 10.1186/s12885-023-11356-6 (PMC10483861; doi:10.1186/s12885-023-11356-6)

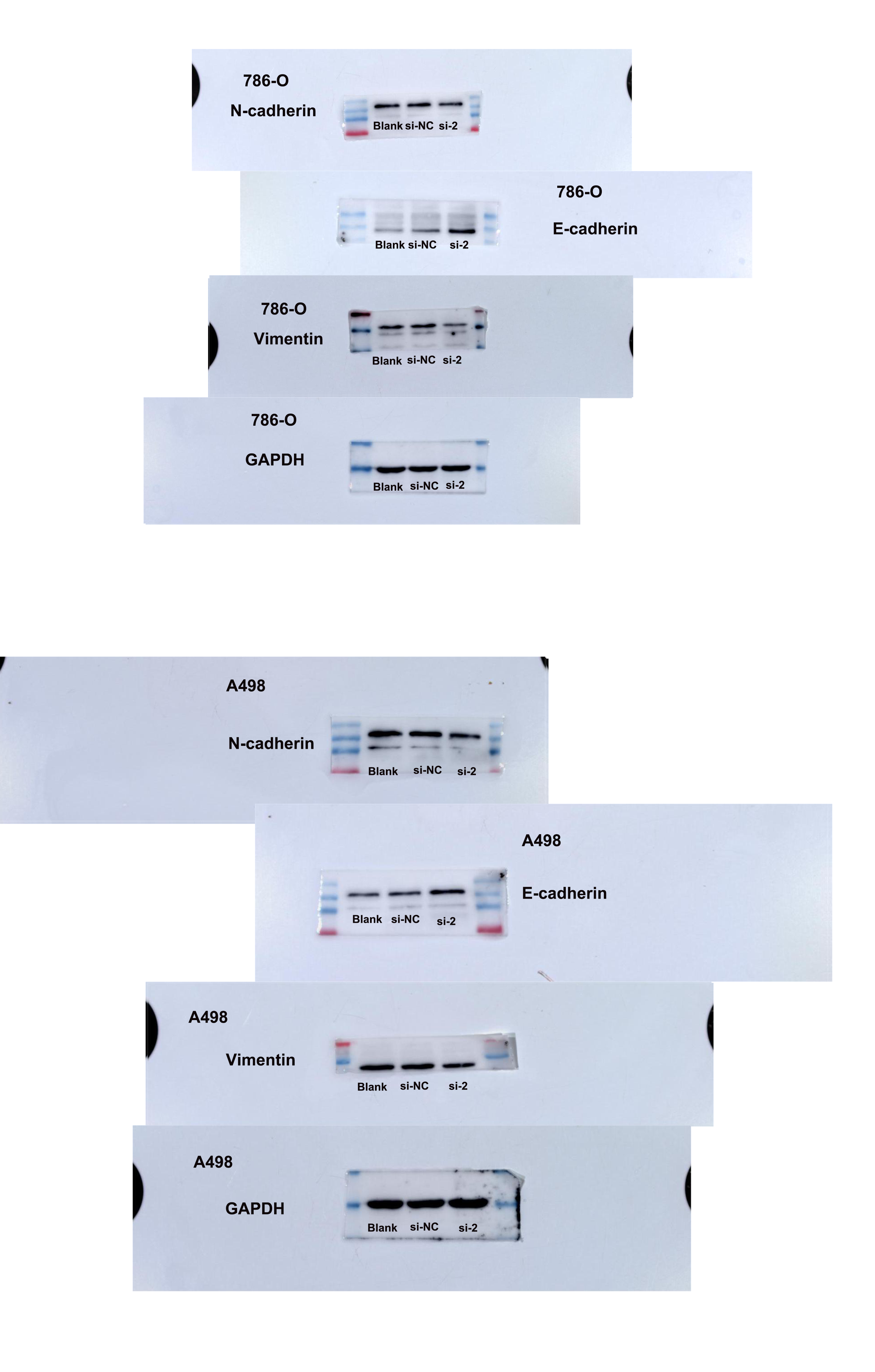

Supplement: Supplementary file 3 — Supplementary Material 3 [file 12885_2023_11356_MOESM3_ESM.tif]
